# Detailed observation on expression dynamics of Polycomb group genes during rice early endosperm development in subspecies hybridization reveals their characteristics of parent-of-origin genes

**DOI:** 10.1186/s12284-019-0306-x

**Published:** 2019-08-13

**Authors:** Quan Kuang, Yinghua Wang, Shisheng Li

**Affiliations:** 1 Department of Biology, Institute of Biotechnology, Nanchang Normal College, Nanchang, 330032 China; 2 Collaborative Innovation Center for the Characteristic Resources Exploitation of Dabie Mountains, Key Laboratories of Economic Forest Germplasm Improvement and Comprehensive Resources Utilization of Hubei province, College of Biology and Agricultural Resource, Huanggang Normal University, Huanggang, 438000 China; 3 College of Software, East China Jiao Tong University, Nanchang, 330013 China

**Keywords:** Polycomb group genes, Rice, Imprinting gene

## Abstract

**Background:**

Parent-of-origin gene expression and its role in seed development have drown a great attention in recent years. Genome-wide analysis has identified hundreds of candidate imprinted genes, a major type of parent-of-origin genes, in rice hybrid endosperms at the stage of 5 days after pollination (dap). However, the expression of these genes in early endosperm have been never confirmed due to technique limitations and the behavior of the imprinted genes in different rice hybridizations are still largely unknown.

**Results:**

Here, based on our elaborate technique established previously, the expression patterns of *PcG* genes in the early stages of endosperm development (within 3 dap), were comprehensively analyzed. We revealed that the free nucleus stage of endosperm development is critical for parent-of-origin gene analysis. The expression of the imprinted genes are highly dynamic, likely corresponding to the critical developmental events during this period. Hybridizations between *Oryza sativa* japonica and indica showed that the expression patterns of the same imprinted gene could be varied by crossing with different parental cultivars, indicative of their parent-dependent character. There are strong alleles that often showed predominant expression over other alleles regardless of the parental origin, which provides a possible explanation for the cultivar-dependent predominant phenotype in crop hybridizations. In addition, we found that the transcripts of the same gene behave differently, with imprinting or non-imprinting patterns, suggesting the existence of not only imprinted and non-imprinted genes but also imprinted or non-imprinted transcripts, which reveals new aspects of the genomic imprinting.

**Conclusions:**

These findings on the characters of parent-of-origin genes shed light on the understanding the real role of gene imprinting in endosperm development.

**Electronic supplementary material:**

The online version of this article (10.1186/s12284-019-0306-x) contains supplementary material, which is available to authorized users.

## Background

Genomic imprinting is a universal epigenetic phenomenon evolved independently in animals and plants, which results in the biased expression of mono allele dependent on their parent-of-origin. In animals, a subset of imprinted genes have been identified and considered to be involved in the regulation of nutrient transfer from maternal tissue to embryo for embryo development (Frost and Moore 2010; Reik et al. 2003; Tycko and Morison 2002; Wood and Oakey 2006). In plants, although several imprinting genes have been identified in early embryos, genomic imprinting are primarily confined to the triploid endosperm, a transient tissue nourishing the developing embryo as placenta in animals. A series of imprinting genes have been identified in endosperms from *Arabidopsis thaliana*, *Oryza sativa* and *Zea maize* through a genome-wide survey (Guo et al. 2003; Hsieh et al. 2011; Luo et al. 2011; Zhang et al. 2011b). Among them, Polycomb Group (*PcG*) genes were explored more intensively due to their important roles in early endosperm development (Hermon et al. 2007; Ingouff et al. 2005; Kinoshita et al. 1999; Nallamilli et al. 2013; Yadegari et al. 2000).

In *A. thaliana*, *PcG* genes were shown to play critical roles in several important transition phases (e.g. from gametophyte to sporophyte), coordinating the development of endosperm, embryo proper and surrounded maternal tissues. Among them, two core components of PRC2 complex, *MEA* and *FIS2*, were revealed as imprinted genes and only transcribed from the maternal allele in endosperm (Kinoshita et al. 1999; Luo et al. 2000). The SET domain of MEA interacts directly with FIE to form a PcG complex, which is crucial for endosperm formation by controlling the activity of a number of imprinted genes in the endosperm (Baroux et al. 2006; Kohler et al. 2005; Spillane et al. 2000; Wang et al. 2006).

In *O. sativa*, great attention has also been paid on the *PcG* genes in endosperm due to the contribution of endosperm to the quality and yield of rice production. Six *PcG* genes including *OsFIE1, OsFIE2, OsEMF2a, OsEMF2b, OsCLF, OsiEz1* have been identified according to the sequence similarity to known *PcG* genes (Luo et al. 2009). The transcripts of all *PcG* genes could be detected in endosperms. Among them, *OsFIE1* and *OsFIE2* were shown to play critical roles in endosperm development (Folsom et al. 2014; Li et al. 2014; Nallamilli et al. 2013). Overexpression of *OsFIE1* result in the precocious cellularization and reduced seed size, whereas down regulation of *OsFIE1* expression resulted in the reduced fertility and delayed embryo development (Folsom et al. 2014). *OsFIE2* has also been reported to play essential roles in the regulation of rice vegetative and reproductive development, especially in the endosperm development and grain filling (Li et al. 2014).

However, whether these developmental defects in endosperm are attributed to the imprinting effect of *PcG* genes is still largely unknown. *OsFIE1* was shown as a maternally expressed imprinting gene in 5 days after pollination (dap) hybrid endosperms from the cross between Nipponbare and IR64 (Luo et al. 2009). And both maternal and paternal transcripts of other five *PcG* genes (*OsiEZ1*, *OsCLF*, *OsEMF2a*, *OsEMF2b*, and *OsFIE2*) in 5 dap hybrid endosperms could be detected simultaneously (Luo et al. 2009). Since several elaborated events of endosperm development such as the formation of primary endosperm nucleus, the initiation of primary endosperm nucleus division and the onset of endosperm cellularization usually occur in the early stage before 5 dap (Brown et al. 1996), and the expression of imprinted genes may be developmental-stage-dependent, it is necessary to screen and confirm the imprinting pattern of these *PcG* genes in very early stages of endosperm development to figure out the role of the imprinted *PcG* genes in seed development. Here, the imprinting pattern of *PcG* genes in the entire process of endosperm development, especially in the early stages of endosperm development (within 3 dap), were analyzed. In addition, the influences of parental genetic background, alterative splicing form on the expression of parental alleles were also discussed in the present study.

## Results

### Identification of DNA polymorphisms of *PcG* genes among Nipponbare, 9311 and Zhonghua 11

**Fig. 1:**
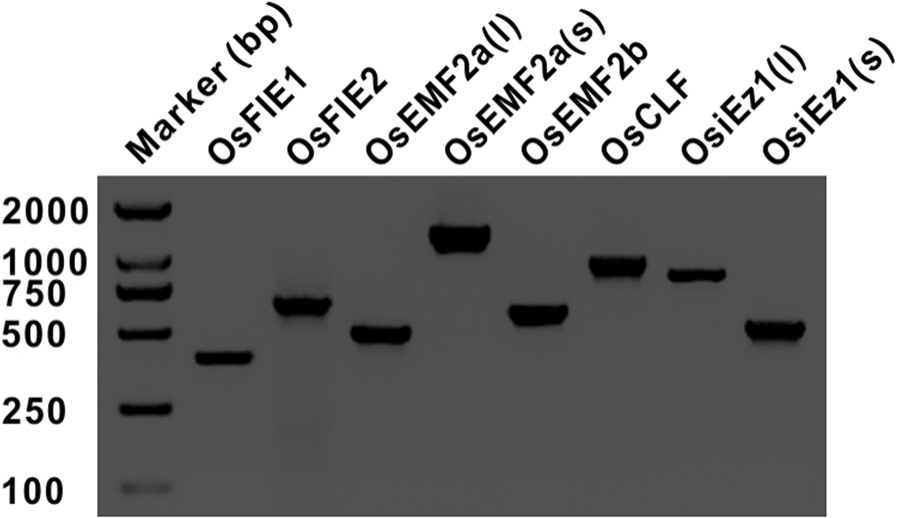
The expression of *PcG* genes in endosperm confirmed by RT-PCR. Distinction between *OsEMF2a(l)* and *OsEMF2a(s)* based on different amplification primers and sequencing primers (See Additional file 1: Table S1)

To explore which allele of *PcG* gene in endosperm was transcribed, the expression pattern of each *PcG* gene in endosperms at different stages (from 1 dap to 7 dap) were carefully examined. The endosperm at different stages were isolated according to our previous method (Kuang et al. 2015). cDNA was prepared using mRNA extracted from endosperm at different stages. RT-PCR analysis results revealed that the transcripts of all *PcG* genes could be detected in endosperms from 1 to 7 dap (Detection results from 3 dap endosperm shown in Fig. 1), giving a possibility to elucidate the dynamics of the expression of parental alleles in endosperms at different stages.

**Fig. 2:**
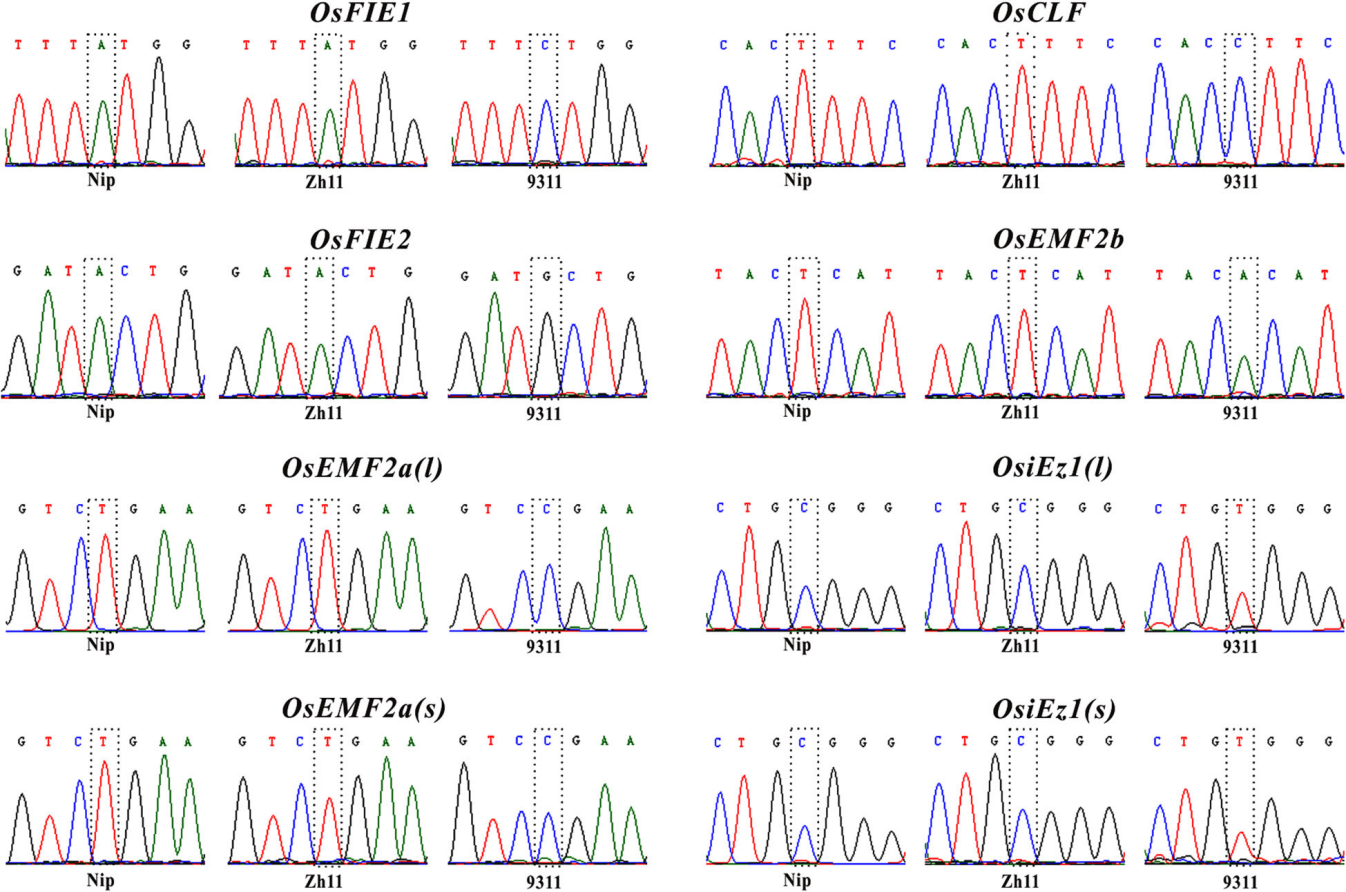
Identification of single nucleotide polymorphisms (SNPs) of *PcG* genes among Nip, 9311 and Zh11. SNPs for each transcript was labeled with dashed box

To distinguish two alleles of *PcG* genes in the genome, the DNA polymorphisms among three cultivated species including Nipponbare (Nip), 9311 and Zhonghua 11 (Zh11) were investigated firstly. The genomic sequences of each *PcG* gene were confirmed by the specific PCR amplification and subsequent Sanger sequencing. Comparing the *PcG* gene sequences from Nip, 9311 and Zh11, the single nucleotide polymorphisms (SNPs) were identified (Fig. 2 and Table 1). There is at least one base polymorphism located in the exon of each *PcG* gene between two subspecies, which could be used to distinguish parental transcript efficiently.

### *OsFIE1* is a maternally expressed imprinting gene at 7 DAP endosperm

**Fig. 3:**
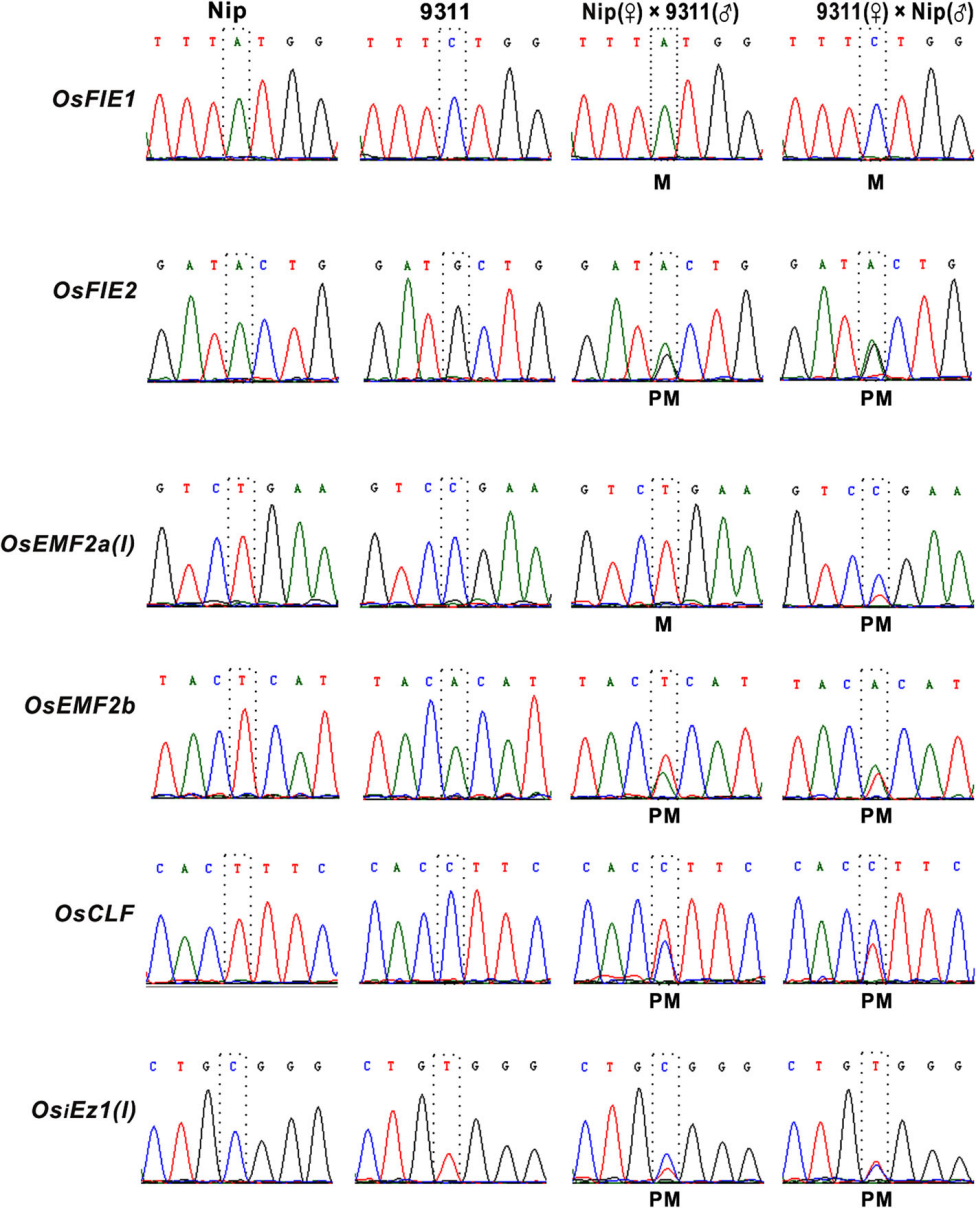
*OsFIE1* is a maternally expressed imprinting gene at 7 DAP endosperm. SNPs for each transcript was labeled with dashed box. Each data has been confirmed by three independent experiments

Early allelic specific expression pattern analysis *PcG* genes in rice endosperm revealed that only *OsFIE1* is maternally expressed imprinting gene among six *PcG* genes (Luo et al. 2009). To confirm allelic expression patterns of *PcG* genes in endosperm, three independent 7 dap hybrid endosperm cDNAs by crossing the Nip and the 9311 reciprocally were produced. Each cDNA sample from hybrid endosperm was subsequently used to amplify the SNP-containing sequence of each *PcG* transcript by RT-PCR with primer sequence flanking SNP. The PCR products were sequenced by Sanger sequencing and assessed for their parent-of-origin biased expression according to SNPs. Then, allele-specific expression analyses of *PcG* genes in endosperm were performed as shown in Fig. 3. Among six *PcG* genes, only *OsFIE1* showed monoallelic expression, confirming that *OsFIE1* is a maternally expressed imprinting gene (Luo et al. 2009). Four *PcG* genes show biallelic expression in 7 dap endosperm, while *OsEMF2a* display either maternal expression pattern or a biallelic expression pattern depending on the direction of the cross between Nip and 9311.

### Dynamics of allele-specific expression of *PcG* genes during endosperm development

Early researches indicated that several key endosperm developmental events including the onset of primary endosperm nucleus division, the compartmentalization of endosperm nuclei and the initiation of endosperm cellularization occur within 3 dap (Brown et al. 1996; Kuang et al. 2015). In addition, the effect of imprinting gene on endosperm development in *A. thaliana* is majorly on early endosperm development (Baroux et al. 2006; Jullien et al. 2006; Luo et al. 2000; Yadegari et al. 2000). Hence, to analyze allelic expression patterns of *PcG* genes comprehensively, hybrid endosperms were isolated at six key stages of endosperm development including stage 1 (1 dap), stage 2 (2 dap), stage 3 (3 dap), stage 4 (7 dap), stage 5 (10 dap) and stage 6 (15 dap), and cDNA samples from hybrid endosperms were prepared according to the previous protocol (Kuang et al. 2015).

**Fig. 4:**
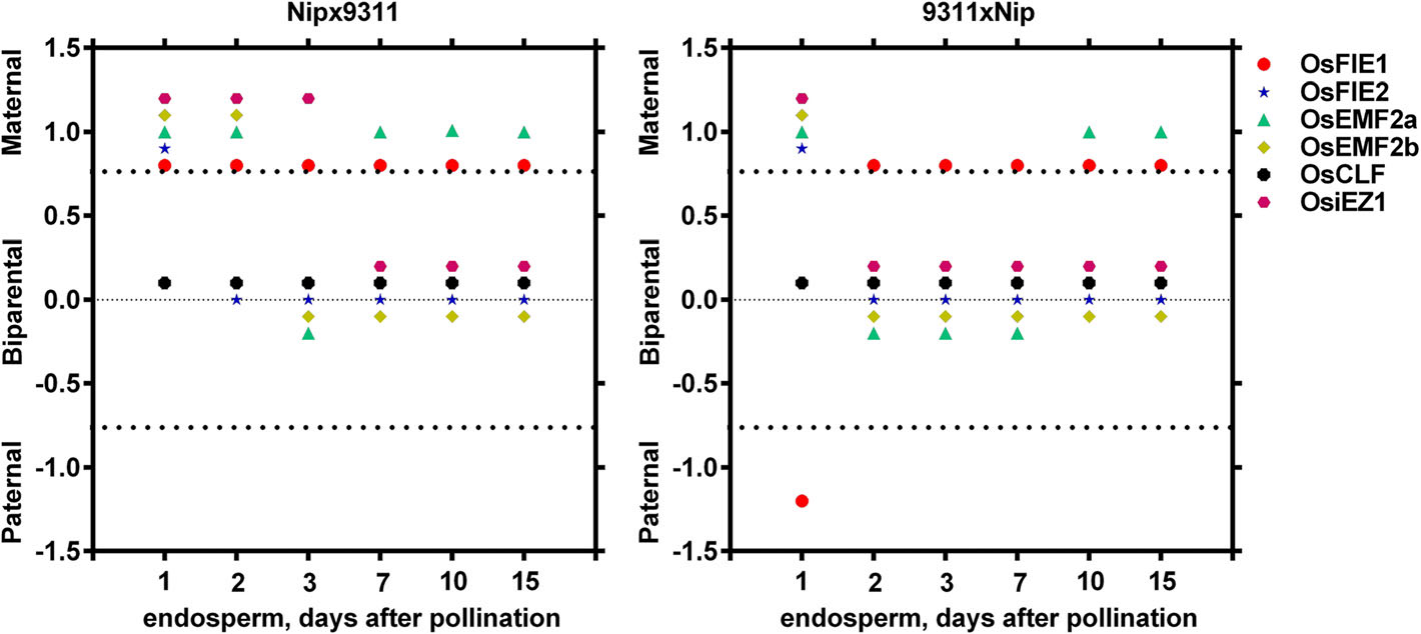
Dynamics of allele-specific expression of *PcG* genes during endosperm development. The dots above the first dash line indicated only transcripts derived from maternal allele could be detected. The dots nearby the middle dash line indicated both transcripts from paternal allele and maternal allele could be detected simultaneously. The dots below the third dash line indicated only transcripts derived from paternal allele could be detected. Each data has been confirmed by three independent experiments

Allele-specific expression analyses of *PcG* genes in the process of endosperm development revealed that the expression pattern of two alleles of *PcG* genes show dynamic changes as endosperm development, especially within 3 dap. Among six *PcG* genes, only *OsCLF* display a stable expression pattern, both transcripts from paternal allele and maternal allele could be detected in endosperms at six stages, indicating *OsCLF* is not an imprinting gene in rice endosperm at these stages tested (Fig. 4). However, parent alleles of other five *PcG* genes show diverse transcriptional activities in endosperms at different stages, especially in stage 1 and stage 2, demonstrating a stage-specific manner. At stage 1, only maternal allele of four *PcG* genes (*OsFIE2*, *OsEMF2a*, *OsEMF2b* and *OsiEZ1*) had been transcribed its transcripts.

In more details, maternal allele of *OsFIE1* was always expressed in endosperm from 1 to 15 dap in Nip × 9311. By contrast, paternal allele of *OsFIE1* was only expressed at 1 dap endosperm in 9311 × Nip. Maternal allele of *OsFIE2* was first expressed in 1 dap endosperm and then parental alleles of *OsFIE2* were both expressed in endosperm at following stages in both Nip× 9311 and 9311 × Nip. More interestingly, *OsEMF2a* was maternally expressed in early and late endosperm, while paternal allele of *OsEMF2a* were only expressed in the middle stage in Zh11 × 9311.With similar expression pattern as *OsFIE2*, both *OsEMF2b* and *OSiEZ1* were maternally expressed in early endosperm and were parentally expressed in following stages. All these data implied that endosperm at free nucleus stage is critical for parent-of-origin gene analysis in rice and the expression of the imprinted genes are highly dynamic or stage-dependent.

### The imprinting pattern of *PcG* genes are influenced by genetic background

**Fig. 5:**
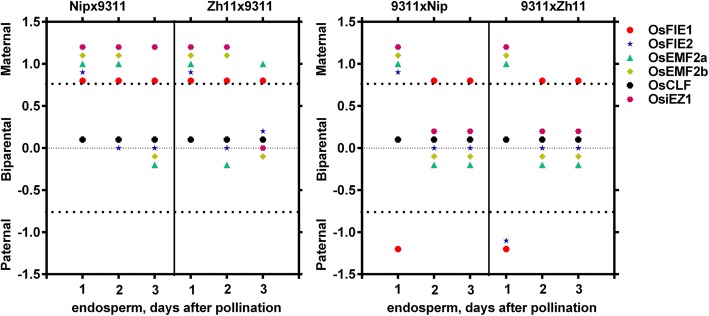
Influence of genetic background on allele-specific expression of *PcG* genes. The dots above the first dash line indicated only transcripts derived from maternal allele could be detected. The dots nearby the middle dash line indicated both transcripts from paternal allele and maternal allele could be detected simultaneously. The dots below the third dash line indicated only transcripts derived from paternal allele could be detected. Each data has been confirmed by three independent experiments

As mentioned above, parent alleles of *PcG* genes display different transcriptional activities based on the direction of the cross between Nip and the 9311, suggesting that the imprinting pattern of *PcG* genes are influenced by different parent combinations. To address this hypothesis, the imprinting pattern of *PcG* genes in two reciprocal cross combinations (Nip or Zh11 with 9311) was compared. The results revealed that the *PcG* genes exhibit notable variation in parental allele activation in the process of endosperm development and the transcriptional activities of two alleles of *PcG* genes are different dependent on the combination of two parents. Among six *PcG* genes, *OsCLF* showed a consistent bi-allelic expression pattern in endosperms in two independent reciprocal cross combinations. Maternal allele of *OsEMF2b* was firstly activated in endosperms (within 2 dap), and parental allele was activated in 3 dap endosperm subsequently. However, other *PcG* genes display notable variation in parental allele activation in two different reciprocal cross combinations. For example, only *OsiEZ1* transcript transcribed from the maternal allele could be detected in 3 dap hybrid endosperm derived from the cross between Nip and 9311, but the transcripts both from maternal allele and paternal allele could be detected simultaneously in 3 dap hybrid endosperm derived from the cross between Zh11 and 9311 (Fig. 5). In addition, the maternal allele of *OsFIE1* was expressed at 1 dap in Zh11 × 9311, while paternal allele of *OsFIE1* was also expressed in 1 dap endosperm 9311 × Zh11. Similar expression pattern of *OsFIE2* is also observed at 1 dap in both Zh11 × 9311 and 9311 × Zh11. Interestingly, in Nip× 9311 reciprocal crosses, it was maternal allele of *OsFIE2* expressed at 1 dap, whereas, in Zh11 × 9311 reciprocal crosses *OsFIE2* seems a dominant allele at 1 dap and associated with Zh11 whenever Zh11 was used as paternal or maternal cultivar. These results indicated the expression patterns of certain gene could be varied by crossing with different parental cultivars and this dominant expression of the imprinted genes associated with specific cultivar is also stage-dependent.

### Different alternative splicing forms of *PcG* genes display different imprinting pattern

**Fig. 6:**
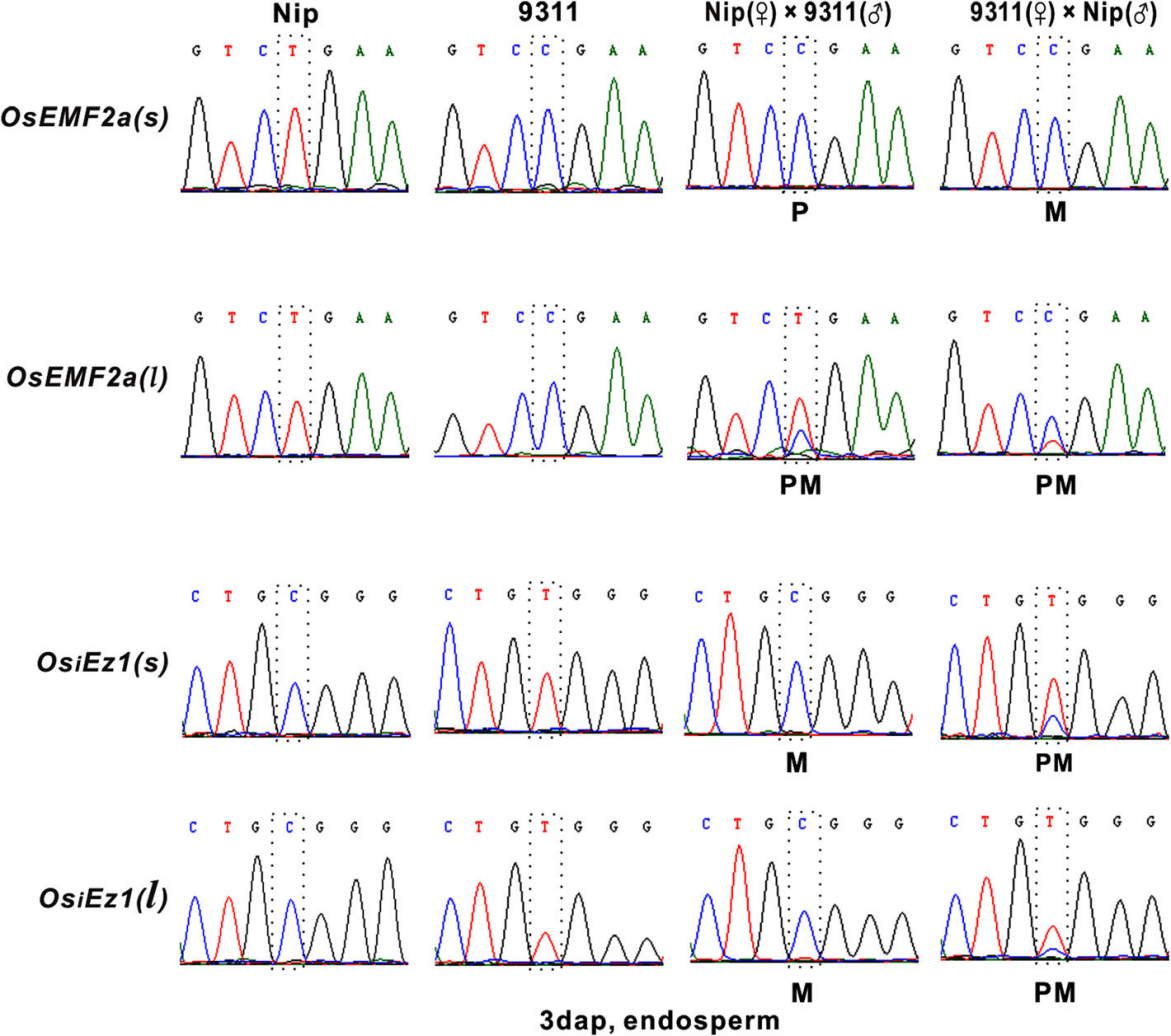
Different alternative splicing form of *PcG* genes display different imprinting pattern. SNPs for each transcript was labeled with dashed box. Each data has been confirmed by three independent experiments

Alternative splicing is an important gene regulatory mechanism in eukaryotes, which results in a single gene coding for multiple proteins. In plants, about least 20% multi-exon genes are alternatively spliced (Estrada et al. 2015). However, whether the two alleles of different alternative splicing forms display similar imprinting pattern is still largely unknown. To address this question, two representative *PcG* genes (*OsEMF2a* and *OsiEZ1*) with distinguishable splicing forms were selected to analyze the imprinting pattern of different splicing forms. *OsEMF2a* and *OsiEZ1* could generate different splicing isoforms with different size. In order to distinguish alternative splicing forms more efficiently, two representative splicing forms, the longest *(l)* and the shortest *(s)* transcripts of *OsEMF2a* and *OsiEZ1,* were selected to analyze the expression of two alleles (Fig. 2 and Table 1). The results revealed that *OsiEZ1(l)* and *OsiEZ1(s),* two alternative splicing forms *OsiEZ1* displayed the same allele-specific expression pattern in the 3 dap endosperm from reciprocal crosses between Nip and 93–11 (Fig. 6). However, two isoforms of *OsEMF2a* display different parent-of-origin expression pattern in the 3 dap endosperm (Fig. 6), providing a clear example of parent of origin-biased transcript isoforms arising from the same gene.

## Discussion

### Uniparental expression pattern of *PcG* genes is developmental stage-dependent during endosperm formation

One clear finding in the present study is that uniparental expression pattern of *PcG* genes displays a developmental stage-dependent manner. *OsFIE1* is only a maternally expressed imprinting gene in the whole process of hybrid endosperm (Nip × 9311) development, and no biased expression of *OsCLF* alleles was found in any developmental stages of hybrid endosperm (Nip × 9311). However, the activation or silencing of two alleles of other four *PcG* genes shows dynamic changes at different stages of endosperm development, suggesting that the expression pattern of parental-origin genes changes as endosperm development, especially in early stages of endosperm development. Similar stage-specific manner of parental-origin genes has also been reported in hybrid embryos. In tobacco, only transcripts from paternal allele of *EB426694* and *CN744644* could be detected in hybrid zygote, whereas both paternal and maternal transcripts could be detected simultaneously in eight-celled embryo (Zhang et al. 2011a). In maize, allele-specific expression assays of 90 genes in endosperms at different stages revealed that only eight of them exhibited persistent maternally or paternally biased expression at multiple stages of endosperm development (Stupar et al. 2007). In *A. thaliana*, only paternal allele of *FUSCA3* was activated at the 2–4 cell embryo stage, but either maternal or biallelic was activated at the globular embryo stage depending on the direction of the cross between *Columbia-*0 (Col-0) and *Landsbergerecta* (Ler) (Raissig et al. 2013). These data revealed that expression pattern of allele specific genes both in embryo and endosperm display a developmental stage-dependent manner, and thus parental and maternal transcripts may have specific contribution to the embryo or endosperm development at specific stage.

Endosperm development in rice experience several critical early stages, including the fusion of the sperm cell with central cell, first cell division of primary endosperm nucleus or promotion of endosperm development, free nuclear movement and oriented distribution, the initialization of cellularization. The molecular mechanism to regulate these critical stages are not well understood. We speculate that there was a correlation between gene imprinting and these critical developmental events. Different imprinted genes may involve in a specific developmental event, not all these events. In addition, parents may differentially contribute to the same developmental events. That’s probably why their expression is highly dynamic and in a stage-dependent manner.

### Alternative splicing forms of a certain transcript displays different expression pattern

Another interesting question raised in the present study is that alternative splicing forms of *PcG* genes display different imprinting patterns. The imprinting pattern of two splicing forms of *OsEMF2a* and *OsiEZ1* were compared in the experiment. Most obviously, the short transcript for *OsEMF2a* (AK069556) is uniparental expressed, but the long transcript (AK120470) is biparental expressed in hybrid endosperm. A similar phenomenon has also been found in inter-subspecies hybrid mice. Allele-specific polyadenylation sites were found at a novel murine imprinted gene (*H13*). Maternal allele preferentially produces the long transcripts, whereas the truncated transcripts are preferentially originated from paternally derived alleles (Wood et al. 2008). This indicates the different roles of the same gene as biparental expressed gene or imprinted genes. Different parentally biased isoforms could generate from same gene via alternative splicing, providing a new aspect of genomic imprinting. The same gene could play both imprinted and non-imprinted roles by produce different transcripts. In this case, it is difficult to name it as an imprinted gene or non-imprinted gene, probably, to be more accurate, we may describe it as imprinted transcript or non-imprinted transcript of the gene. This finding enhances our understanding of the complexity of genomic imprinting.

### Uniparental expression pattern of *PcG* genes is influenced by parental genetic background

It was reported that it will suffer a very important effects to the offspring’s phenotypic and quality traits by using different male and female parents as hybrid material, or exchanging parents’ position for reciprocal cross. This phenomenon is usually considered to be the result from unbalanced dose ratio between maternal genome and paternal genome during the reciprocal-crossing processes (Dilkes et al. 2008; Scott et al. 1998). Our work provides new explanations for the phenomenon. The uniparental expression pattern of genes is obviously influenced by parental genetic background. Some imprinted genes of certain cultivars could predominantly expresses in the hybrids whenever the cultivar is used as maternal or paternal material in the crosses, indicating that the behavior of the imprinted genes could be associated with certain genotype. The regulatory mechanism underlying the phenomenon is interesting but not yet understood. However, it is clear that the imprinting effects of the genes will certainly influence the phenotype, e.g. endosperm development, therefore, it may enable some specific cultivar with superiority to control specific phenotypes or developmental characters in hybrids. Thus, it might be an alternative explanation for the parent-associated characters in crop crosses and provides new clue for the selection of the parents in crop breeding.

## Methods

### Plant materials

Three *Oryza sativa* species including two *Japonica cultivars* Nipponbare, Zhonghua 11 and one *Indica cultivar* 9311 were used in the present study, which were cultivated in the greenhouse with 13 h of illumination every day. The daytime temperature was 30 °C, and the night temperature was 25 °C.

### Polymorphism detection between rice species

In order to detect DNA polymorphisms among Nip, 9311 and Zh11, the genomic sequences of *PcG* genes (*OsFIE1, OsFIE2, OsEMF2a, OsEMF2b, OsCLF, OsiEz1*) were downloaded from rice genomic database, which is from “http://www.ricedata.cn/gene/” or “https://rapdb.dna.affrc.go.jp/” in this experiment. In the meantime the sequences of *PcG* genes from Nip, 9311 and Zh11 were confirmed through PCR, respectively. And the genomic sequences of *PcG* genes from two different species were aligned and compared to detect DNA polymorphisms.

### RNA extraction and RT-PCR

The collection of hybrid ovaries at the same stages, endosperm isolation and mRNA extraction were carried out according to the previous protocol (Kuang et al. 2015). For the crosses, the flowers were previously labeled, emasculated, and hand-pollinated. After emasculation and pollination, the flowers were covered by paper bag, therefore, self-pollination was totally excluded and the time after pollination is exactly controlled. The detection on SNP loci in *OsCLF* ensured the hybrid ovaries for the experiments. cDNA were synthesized using SuperScript III Reverse Transcriptase (Thermo fisher Scientific) under the conditions recommended by the manufacturer. RT-PCR was performed in a 20 μl PCR mixture containing 2 μl of 10× Ex *Taq* buffer (including Mg^2+^), 200 μM dNTPs, 0.2 μM of primers, 0.5 U Ex *Taq* DNA polymerase (Takara), and cDNA prepared from endosperms at different stages. PCR conditions are performed as follows: initial denaturation at 94 °C for 2 min; 40 amplification cycles with denaturation at 94 °C for 30 s, annealing at 56 °C for 30 s; extension at 72 °C for 1 min; and a final incubation at 72 °C for 5 min. Each PCR products were purified and sequenced to distinguish which allele was transcribed according to DNA polymorphisms.

For genes with multiple transcripts such as *OsEMF2a*, we designed primers on the specific sequences of different regions from different transcripts, which can amplify specific splices of different lengths (Table 1). In general, long transcripts can translate proteins, and short transcripts cannot translate proteins without initiation codon ATG.

**Table 1:**
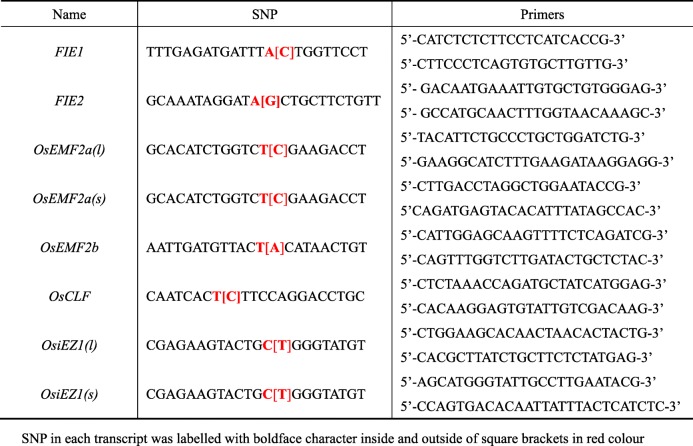
SNPs used to distinguish paternal and maternal transcripts in endosperm

## Additional file

Additional file 1: **Table S1.** Primers of Distinction between OsEMF2a(l) and OsEMF2a(s). (DOC 27 kb)

## Data Availability

The datasets supporting the conclusions of this article are included within the article.

## Abbreviations

9311: *Oryza sativa subsp. indica* (9311)
DAP/dap: Days after pollination
Nip: *Oryza sativa subsp. japonica* (Nipponbare)
PcG: Polycomb group
PCR: polymerase chain reaction
PRC2: Polycomb Repressive Complex 2
RT-PCR: Reverse transcriptase polymerase chain reaction
SNPs: Single nucleotide polymorphisms
Zh11: *Oryza sativa subsp. japonica* (Zhonghua 11)
