# Supplementary material for: Detailed observation on expression dynamics of Polycomb group genes during rice early endosperm development in subspecies hybridization reveals their characteristics of parent-of-origin genes
Source: Rice (N Y). 2019 Aug 13;12:64. doi: 10.1186/s12284-019-0306-x (PMC6692421; doi:10.1186/s12284-019-0306-x)
Supplement: Supplementary file 1 — Table S1. Primers of Distinction between OsEMF2a(l) and OsEMF2a(s). (DOC 27 kb) [file 12284_2019_306_MOESM1_ESM.doc]

**Table S1. Primers of Distinction betweenOsEMF2a(l) and -OsEMF2a(s)**

| Name | SNP | Primers | fragment size |
| --- | --- | --- | --- |
| *OsEMF2a(l)* | GCACATCTGGTC**T**[**C**]GAAGACCT  AATATGACCAGG**C[T]**GCAGAAGG | 5’-TACATTCTGCCCTGCTGGATCTG-3’  5’-GAAGGCATCTTTGAAGATAAGGAGG-3’ | 510bp |
| *OsEMF2a(s)* | GCACATCTGGTC**T**[**C**]GAAGACCT | 5’-CTTGACCTAGGCTGGAATACCG-3’  5’CAGATGAGTACACATTTATAGCCAC-3’ | 1402bp |

SNP in each transcript was labelled with red character.
